# Stocking density modulates tilapia (*Oreochromis niloticus*) performance and fillet quality through phospholipid remodeling

**DOI:** 10.1038/s41598-025-25814-7

**Published:** 2025-11-25

**Authors:** Gabriel Roldi, Samuel Robath, Carla Porto, Eduardo Pilau, Percilia Giaquinto, Joao Favero Neto, Bruno Lala, Gracielle Caroline Mari, Carolina Toledo Santos, Gabriel Naziazeno Anastácio, Gabriela Hernandes Cangianelli, Andre Lima Ferreira, Caio César Ouros, Gustavo Henrique Coelho Chavesk, Maria Luiza Rodrigues Souza, Diogo de Oliveira Marques, Stefania Caroline Claudino-Silva

**Affiliations:** 1 Maringá, Paraná Brazil; 2 Provincial Directorate of Science and Technology, Higher Education and Technical-Vocational Training of Niassa, Maputo, Mozambique; 3 Department of Chemistry, State University of Maringá (UEM), Maringá, Paraná Brazil; 4 MS Bioscience, Maringá, Paraná Brazil; 5 CAUNESP – Aquaculture Center of Unesp, São Paulo State University (Unesp), Jaboticabal, SP Brazil; 6 Federal Rural University of the Amazon (UFRA), Belém, Pará Brazil; 7 Department of Animal Science, State University of Maringá (UEM), Maringá, Paraná Brazil; 8 College of Agricultural Sciences of Botucatu (FCA), Botucatu, São Paulo Brazil; 9 São Paulo State University (UNESP), Assis, São Paulo Brazil; 10 Federal Institute of Education, Science and Technology of Goiás (IF Goiano), Research Group On Aquatic Life and Sustainable Production (AQUAVITA), Campos Belos, GO Brazil; 11 Federal University of Grande Dourados, Mato Grosso Do Sul, Brazil; 12 Universitary Center of Goiatuba – UNICERRADO, Goiás, Brazil

**Keywords:** Chronic stress, Lipid metabolism, Phospholipid remodeling, Fillet quality, Aquaculture, Fish welfare, Ecology, Ecology, Physiology, Zoology

## Abstract

This study evaluated the effects of different stocking densities (high density–HD: 14 fish/tank; low density–LD: 7 fish/tank) on productive performance, lipidomic profile, and fatty acid composition in GIFT tilapia juveniles. Zootechnical parameters were analyzed, including relative growth, weight gain, feed conversion ratio, and fillet yield, along with lipidomic markers and fatty acids. Results showed that high density promoted better apparent feed conversion (*p* < 0.0001). However, low density led to higher apparent feed intake (*p* < 0.0001) and greater fillet yield (*p* = 0.0009), suggesting these fish utilized ingested nutrients to maximize fillet production. Lipidomic analysis revealed higher phospholipid turnover in low density (LD), with increased lysophosphatidylcholines (LPC) and lysophosphatidylethanolamines (LPE), while high density (HD) showed phosphatidylcholine (PC) accumulation, indicating less efficient lipid metabolism under stress. Additionally, LD had higher concentrations of polyunsaturated fatty acids (PUFAs) (*p* < 0.05), highlighting density’s influence on nutritional quality. We conclude that low stocking density suggests an improvement in productive performance and fish quality, whereas high density induces metabolic responses indicative of chronic stress related to lipid metabolism.

## Introduction

Aquaculture has emerged as a key activity for global food security, meeting the growing demand for animal protein in a more sustainable way compared to extractive fishing. Among cultivated species, tilapia stands out as one of the most important, being widely produced in various countries due to its adaptability, rapid growth and high market acceptance^1,2^. In tilapia, stocking density is particularly critical because this species is widely cultivated in intensive and superintensive systems, where high densities are common^3–10^. In addition, their hierarchical social behavior and territorial disputes may intensify competition for food and space under crowded conditions, directly influencing performance and welfare^11–13^. However, to ensure the economic and environmental viability of this activity, it is essential to optimize farming practices, particularly stocking density^1,14^.

At high densities, fish are more susceptible to chronic stress, competition for resources such as food and space, and increased disease incidence, which may compromise meat quality and farming efficiency^10,15,16^. Low densities tend to promote a more balanced environment with less competition, but the lack of adequate social interaction may influence the expression of the species’ natural behavior and produce worse results than those found at high culture densities^6,17^. In this sense, finding markers to evaluate the physiological effects of stocking density becomes essential to optimize management.

Lipids are essential in fish metabolism, serving as energy sources, cellular membrane components, and signaling molecule precursors^18–20^. The regulation of lipid metabolism is intrinsically linked to other critical metabolic pathways and may provide useful indicators for assessing the physiological effects of various stressors on fish. Chronic stress, common in high stocking densities, has been associated with dysregulation of fatty acids, phosphatidylethanolamine (PE), phosphatidylcholine (PC), and lysophosphatidylcholine (LPC) in fish species such as Amur sturgeon (*Acipenser schrenckii*), killifish (*Fundulus heteroclitus*), bluegill (*Lepomis macrochirus*), stone loach (*Triplophysa bleekeri*), largemouth bass (*Micropterus salmoides*), rainbow trout (*Oncorhynchus mykiss*), and Japanese sea bass (*Lateolabrax maculatus*)^21–26^.

In fish, similar lipid metabolism alterations have been observed under stressful conditions, suggesting these evaluations may help elucidate physiological and biochemical mechanisms underlying stress responses^27–29^. The integration of productive performance, fillet quality, and lipidomic profiling provides a more comprehensive understanding of how stocking density affects fish. While performance and fillet quality reveal the practical consequences for aquaculture, lipidomics offers mechanistic insights, identifying molecular signatures that may explain alterations in growth efficiency and meat composition. Lipidomics allows the identification of molecular signatures that would not be revealed by performance and fillet quality indicators alone, and this can open new avenues for aquaculture management. This integrative approach is still scarce in tilapia studies and represents a novel strategy to link physiological mechanisms to productive outcomes. In this context, we hypothesize that different stocking densities modulate lipid metabolism and productive performance, significantly impacting growth and lipid profiles. Therefore, this study aims to evaluate the effects of different stocking densities on (1) productive performance, (2) fillet quality and (3) lipidomic profile in tilapia juveniles.

## Results

### Environmental variables and performance

Water quality variables were maintained within suitable ranges for tilapia culture: temperature (23.8 ± 1.35 °C), pH (6.9 ± 0.2), total ammonia (0.0015 ± 0.001 mg L⁻^1^), and dissolved oxygen (7.8 ± 1.5 mg L⁻^1^) among the hapas. The performance parameters showed significant differences between fish reared at high and low densities (Table 1). Rearing density affected fish metabolism and feed efficiency, leading to changes in nutrient conversion and overall performance. This was reflected in a notably improved AFCR in the HD group (*p* < 0.0001). At low densities, fish showed higher AFI (*p* < 0.0001) and higher FY (*p* = 0.0030), but WG remained unchanged between groups (*p* = 0.7127).

**Table 1:** Performance parameters of juvenile tilapia reared at high (HD) and low (LD) stocking densities.

Parameter	HD (*Means* ± *SD*)	LD* (Means* ± *SD)*	*p*-Value
Apparent feed intake (AFI)	38.40 ± 0.51^b^	46.50 ± 0.54^a^	** < 0.0001**
Weight gain (WG)	30.92 ± 5.99^a^	30.31 ± 6.52^a^	0.7127
Apparent feed conversion ratio (AFCR)	1.29 ± 0.25^b^	1.61 ± 0.38^a^	** < 0.0001**
Fillet yield (FY)	25.95 ± 2.90^b^	28.16 ± 2.16^a^	**0.0030**

*SD* Standard deviation. Different letters in the same row indicate statistical difference by *Student’s t-test* (*p* < 0.05).

Significant values are in bold

### Fillet quality

**Fig. 1:**
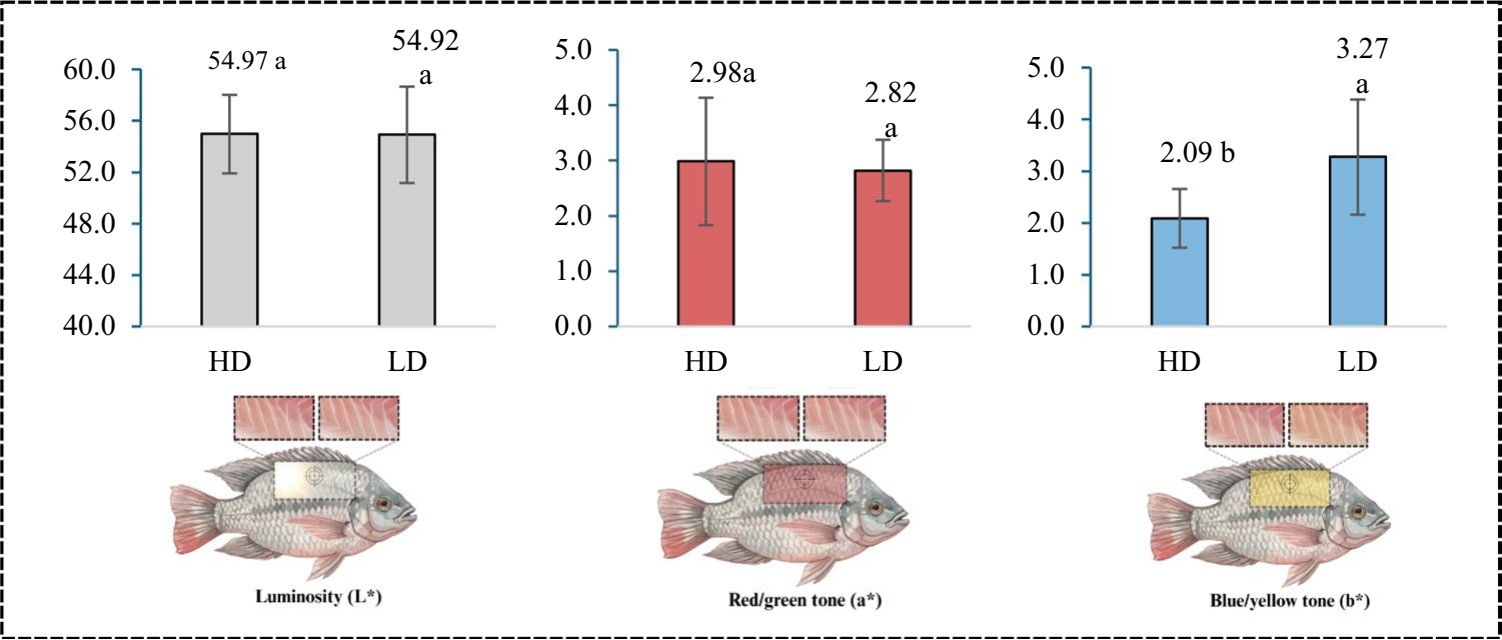
Color parameters (L*, a*, and b*) of Nile tilapia fillets reared at different stocking densities. The schematic diagrams overlaid on the fish indicate the central dorsal region of the fillet used for sample collection, ensuring consistent sampling positions between groups. Representative photographs of fillets from each group (HD = high density; LD = low density) are shown next to the graphs to visually display color differences. Values are expressed as mean ± standard deviation. Different letters above the bars indicate statistically significant differences according to Student’s *t*-test (*p* < 0.05).

Regarding fillet texture, differences emerged between treatments for deformation hardness (mm) (*p* = 0.014), with higher values in the HD group (3.87 ± 0.35) compared to the LD group (3.51 ± 0.1509). However, maximum force (N) recorded during the first fillet compression did not vary significantly between treatments (*p* = 0.6136). No differences were observed in fillet pH between LD and HD groups (6.71 ± 0.18–HD and 6.76 ± 0.07 – LD; *p* = 0.3675). The density levels had no effect on luminosity (L*; *p* = 0.7320) or red/green tone (a*; *p* = 0.9734) but did alter the blue/yellow tone (b*; *p* = 0.0111) (Fig. 1).

### Proximal composition and fat acids content

Rearing conditions did not change moisture, protein, or ash content, but a clear effect was detected in fillet fat content, with higher values in LD-reared fish (Table 2).

**Table 2:** Proximate composition of tilapia fillets reared at high (HD) and low density (LD).

Parameter	HD (Means ± SD)	LD (Means ± SD)	*p*-Value
Moisture (%)	79.86 ± 0.56	79.80 ± 0.98	0.8029
Ash (%)	1.35 ± 0.07	1.33 ± 0.10	0.4917
Crude Protein (%)	17.62 ± 0.53	17.59 ± 1.11	0.9082
Ether extract (%)	1.10 ± 0.07^b^	1.18 ± 0.05^a^	**0.0005**

*SD* Standard deviation. Different letters in the same row indicate statistical difference by *Student’s t-test* (*p* < 0.05).

Significant values are in bold

The fatty acid profile results are presented in Table 3. The LD group contained more short-chain saturated fatty acids compared to the HD group (*p* = 0.0247). No differences were evident between groups for monounsaturated fatty acids (*p* = 0.2124).

**Table 3:** Fatty acid profile comparison between juvenile tilapia reared at high (HD) and low (LD) stocking densities.

	HD *(Means* ± *SD)*	LD *(Means* ± *SD)*	*p-*Value
SATURATED Short-Chain	1.17 ± 0.82b	2.13 ± 0.13a	0.0247
SATURATED Medium-Chain	0.08 ± 0.04a	0.05 ± 0.01a	0.1426
SATURATED Long-Chain	35.84 ± 0.92a	36.43 ± 1.20a	0.3064
Total Saturated	37.09 ± 1.70a	38.61 ± 1.17a	0.0737
MONOUNSATURATED	26.13 ± 3.80a	23.97 ± 2.11a	0.2124
TOTAL PUFA	32.83 ± 3.05b	37.42 ± 1.80a	0.0074
PUFA n-3	3.21 ± 0.05b	3.58 ± 0.29a	0.0173
PUFA n-6	29.62 ± 3.03b	33.83 ± 1.53a	0.0105

*SD* Standard deviation. PUFAs (Polyunsaturated Fatty Acids). Different letters in the same row indicate statistical difference by *Student’s t-test* (*p* < 0.05).

### Lipidomic markers

**Fig. 2:**
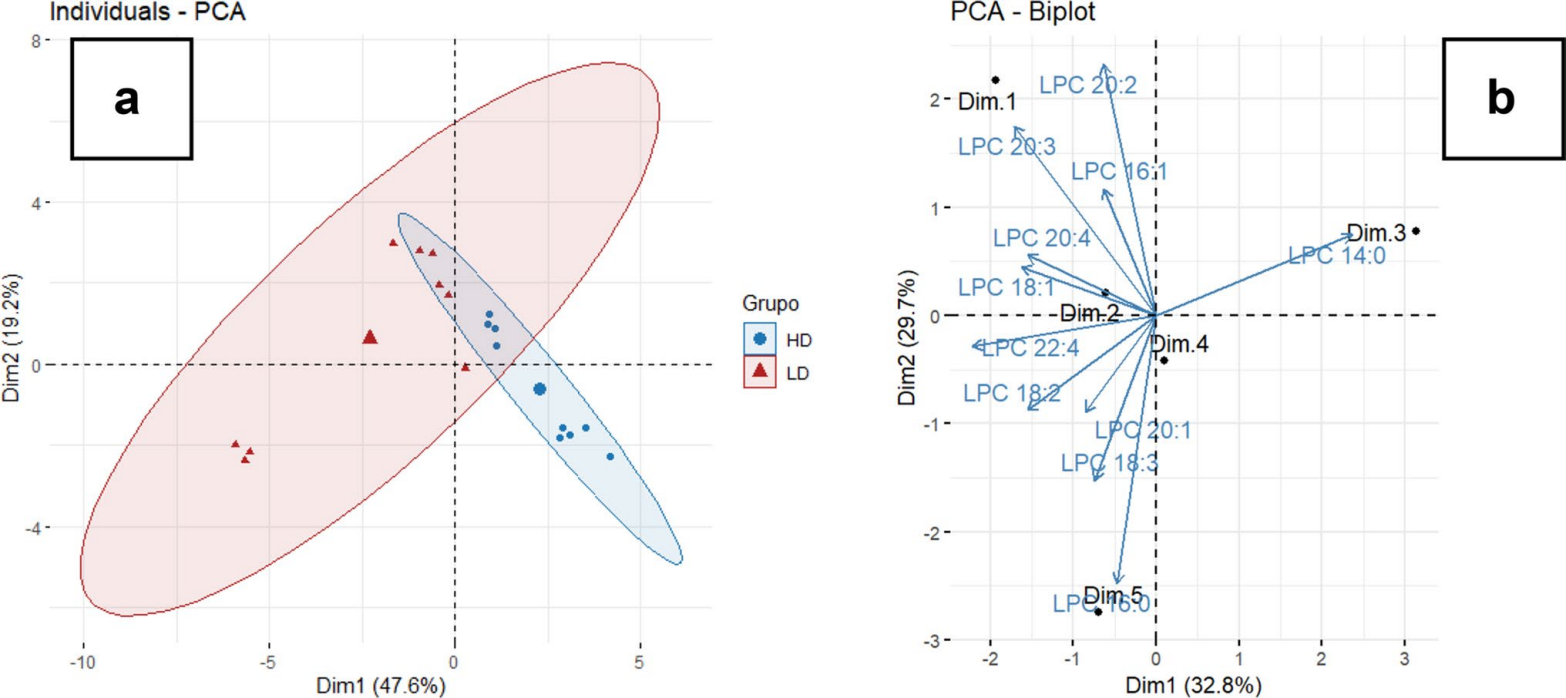
Principal Component Analysis (PCA) of lipidomic data from juvenile Nile tilapia reared at different stocking densities (HD = high density; LD = low density). (**a**) PCA score plot with 95% confidence ellipses for each group, illustrating the degree of separation between HD and LD. (**b**) PCA loading plot (biplot) identifying the phospholipid molecules that contributed most to group separation along the first two principal components.

The lipidomic analysis identified multiple lipid classes, including phospholipids, sphingolipids, glycolipids, cholesteryl esters, bioactive lipids, and other metabolites. Differences in lipid profiles between groups were statistically confirmed by the PERMANOVA test (*p* = 0.001). Principal component analysis (PCA) revealed that the metabolites contributing most to sample discrimination were mainly phospholipids (55%), followed by ethanolamines (20%), glycolipids (20%), and sphingolipids (5%) (Fig. 2). The first two principal components explained 47.6% (Dim1) and 19.2% (Dim2) of the total variance, respectively. Group separation occurred primarily along Dim1, with HD samples clustering toward the negative axis and LD samples toward the positive axis (Fig. 2A). The LD group also exhibited greater within-group dispersion, as shown by the broader spread of points compared to the HD group. The ellipses represent the 95% confidence interval for each group, illustrating both the degree of separation and group variability. The loading plot (Fig. 2B) indicated that this separation was mainly driven by specific phosphatidylcholines (e.g., PC 16:1, PC 20:1, PC 22:4) and lysophosphatidylcholines (e.g., LPC 14:0, LPC 20:3), which contributed most to the variance captured by Dim1 and Dim2.

**Fig. 3:**
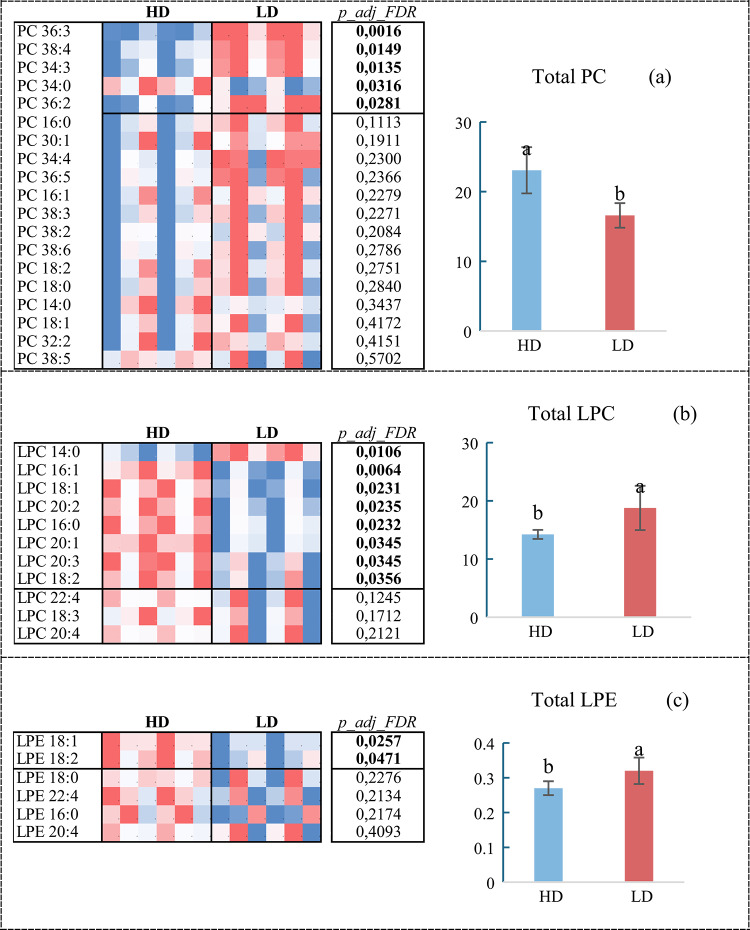
Phospholipid content in juvenile tilapia reared at high (HD) and low (LD) stocking densities. Different letters above the standard deviation bars indicate statistically significant differences by Student’s *t*-test (*p* < 0.05). (**A**) Phosphatidylcholines—PC, (**B**) Lysophosphatidylcholines – LPC and (**C**) Lysophosphatidylethanolamines—LPE. (*p_adj_FDR*: False Discovery Rate (FDR) Adjusted *P*-value).

Initial analysis showed that the different stocking densities did not influence total phospholipids (*p* = 0.4072). However, when analyzing the subclasses, contrasting responses appeared: total PC was higher in the HD group (*p* = 0.0228), while total LPC (*p* = 0.0318) and total LPE (*p* = 0.0286) were higher in the LD group. No statistical differences were found for PE (*p* = 0.1783), PI (*p* = 0.9435), or PC O (*p* = 0.3009). Within the PC category, five compounds were influenced by rearing density (*p* < 0.05), with PC 34:0 being higher in the LD group, while PC 36:2, PC 34:3, PC 36:3, and PC 38:4 were higher in the HD group (Fig. 3a). For LPC, eight compounds were affected (*p* < 0.05), with LPC 14:0 being higher in the HD group, while LPC 16:1, LPC 18:1, LPC 20:2, LPC 16:0, LPC 20:1, LPC 20:3, and LPC 18:2 were higher in the LD group (Fig. 3b). Finally, for LPE, two compounds (LPE 18:1 and LPE 18:2) were influenced by stocking density (*p* < 0.05), both being higher in the LD group (Fig. 3c).

## Discussion

This study demonstrates that stocking density exerts multiscale effects on the performance and fillet quality of Nile tilapia, impacting physical attributes (texture, color), lipid composition, and lipidomic profiles (phospholipids). These differences have direct implications for animal welfare, nutritional and sensory quality of the fillet, and its market value.

The observed differences in production parameters indicate that stocking density directly affects feed efficiency and fillet yield, despite not significantly altering overall weight gain. Although fish reared at low density (LD) consumed more nutrients, their apparent feed conversion was less efficient compared to the high-density (HD) group (*p* < 0.0001). However, nutrients ingested under LD conditions were preferentially allocated to increased fillet yield (*p* = 0.0030). This pattern aligns with findings previously^30^, who reported that juvenile rainbow trout (*Oncorhynchus mykiss*) raised at higher densities reduce feed waste due to increased intraspecific competition, promoting more efficient and targeted consumption.

Some authors suggest that moderate stress induced by high stocking density may modulate energy metabolism, favoring nutrient allocation to essential processes such as homeostasis maintenance^31–33^. This metabolic modulation could explain the superior feed efficiency observed in the HD group. Nonetheless, despite greater efficiency, fillet yield was significantly lower, possibly due to energy reallocation toward maintenance and survival processes at the expense of muscle biomass accumulation.

Under high-density conditions, prioritization of cortisol production and other physiological stress responses can limit somatic growth^8^. Moreover, increased competition for space and resources tends to elevate energy expenditure on swimming and agonistic behaviors, reducing energy available for muscle deposition^34,35^.

In this study, higher deformation hardness was observed in the HD group, whereas maximum force did not differ significantly between groups. This indicates that although the HD muscle exhibited greater elasticity, its resistance to compression remained similar to that of the LD group. These results suggest that the muscle structure in HD fish, despite being more deformable, maintained comparable firmness, possibly due to differences in fiber organization or density without compromising the tissue’s load-bearing capacity.

Regarding coloration, a significant increase in the b* parameter (yellowness) of the fillet surface was detected without marked changes in pH or luminosity (L*). The elevated b* value in the LD group may be associated with a higher lipid content in the fillet, likely due to reduced feeding competition and consequently greater lipid consumption and storage in these fish. This relationship between stocking density and lipid composition has been reported previously^9,36^. Furthermore, the yellowish hue may reflect increased lipid oxidation^37^.

The LD group showed significantly higher levels of polyunsaturated fatty acids (PUFAs) across all subcategories, indicating that low-density conditions favor the synthesis or retention of these lipids essential for fundamental cellular functions^38^. Additionally, increased levels of short-chain fatty acids may reflect enhanced metabolic efficiency and environmental adaptation^39^, who linked these lipids to stress response and energy homeostasis maintenance.

The elevated lipid content observed in the LD group aligns with evidence that high stocking densities impair growth and fat accumulation due to activation of stress-related metabolic responses. For instance, studies on *Acipenser schrenckii* revealed that higher density reduced adipose tissue and lipase expression while upregulating PPARα expression, a key regulator of energy mobilization^22^. These findings corroborate prior reports on lipid alterations induced by environmental stressors^40^ and underscore the critical role of PUFAs in cellular homeostasis^41^.

The lipidomic alterations observed in this study primarily involved the classes of phosphatidylcholines (PC), lysophosphatidylcholines (LPC), and lysophosphatidylethanolamines (LPE). Although the total phospholipid content did not differ between experimental groups, the breakdown of PC species revealed significantly higher levels in the HD group, while LPC and LPE were more abundant in the LD group, indicating a clear dissociation between total phospholipid content and the composition of individual subclasses. The most affected PC species included PC 36:2, PC 34:3, PC 36:3, and PC 38:4, all of which were more abundant in HD, whereas PC 34:0 was higher in LD. These significant alterations in individual species suggest an adaptive compositional remodeling of the membrane^42,43^, rather than a global disruption of lipid homeostasis. This selective response aligns with the eurythermal and highly adaptable nature of tilapia^44–46^, contrasting with the generalized metabolic suppression reported in cold-water species under density stress^25^.

**Fig. 4:**
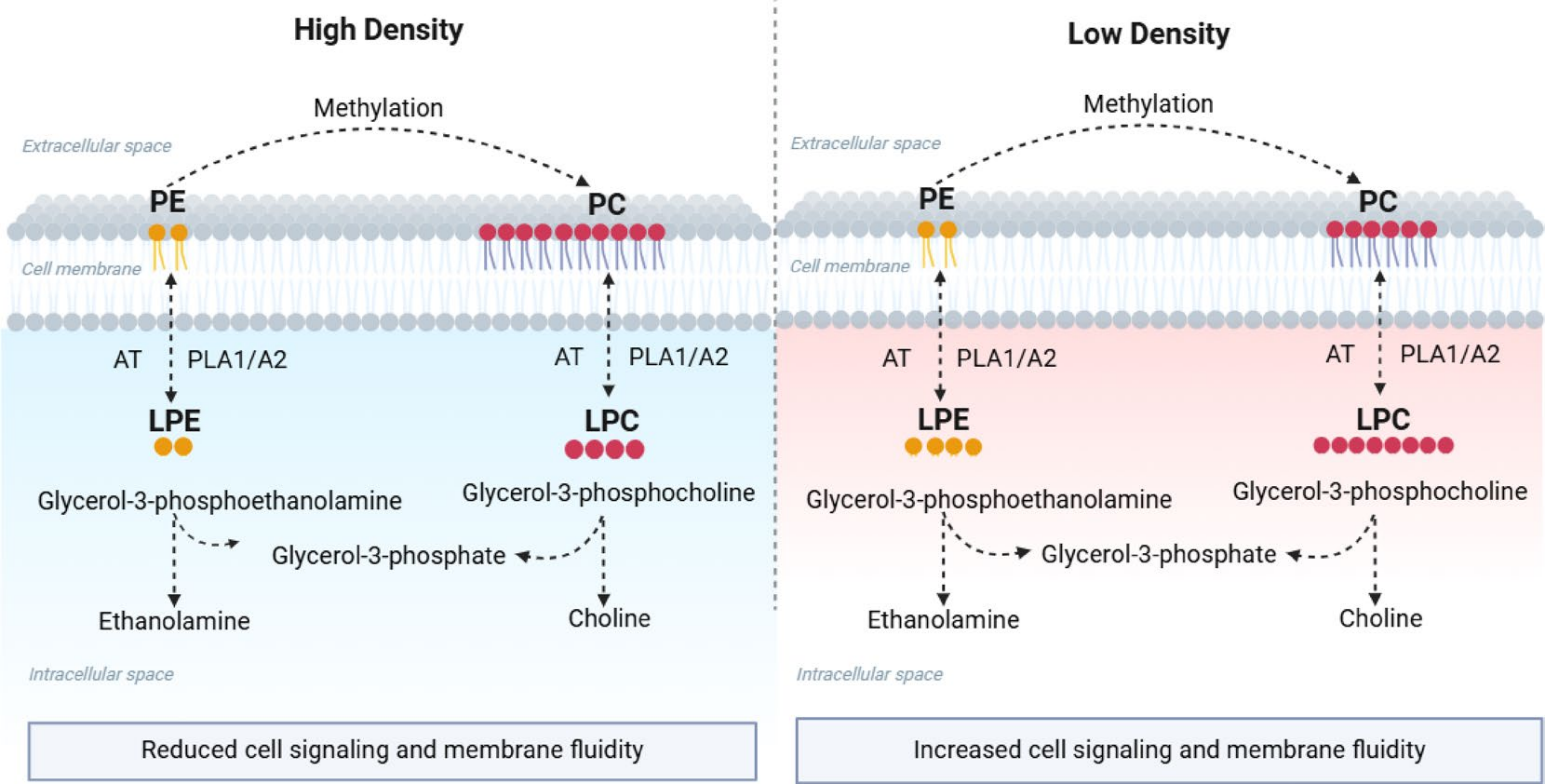
Illustrative scheme of changes in the cellular membrane lipid composition of tilapia reared at high density (HD) and low density (LD). Under high density, there is a higher proportion of Phosphatidylcholine (PC), resulting in a more rigid membrane. Under low density, increased production of Lysophosphatidylcholines (LPC) and Lysophosphatidylethanolamines (LPE) is observed, promoting greater membrane fluidity. The conversion of PE to PC occurs via methylation, while phospholipid degradation is mediated by phospholipases A1/A2 (PLA1/A2), and re-conversion by acyltransferases (AT).

The higher abundance of unsaturated PCs (such as PC 36:3, 34:3, and 38:4) in the HD group may represent a compensatory adaptation aimed at maintaining membrane fluidity^47,48^. This pattern suggests modulation of the Lands cycle, possibly reflecting reduced phospholipase A₂ (PLA₂) activity and/or increased reacylation activity mediated by LPCAT and LPEAT. Although these enzymes were not quantified in the present study, the observed profile is consistent with PLA₂ suppression and a relative increase in the reacylation pathway, as described in other teleosts^49,50^. Under normal conditions, PLA₂ catalyzes the hydrolysis of phospholipids, releasing a fatty acid and generating LPC and LPE, while LPCAT and LPEAT reconvert these molecules into PCs and PEs, respectively, through reacylation reactions^49–51^ (Fig. 4).

Thus, a decrease in LPCs concomitant with an increase in PCs may suggest reduced phospholipid turnover and a higher PC/(LPC + LPE) ratio, indicating decreased membrane lysis and greater structural stability of the bilayer—plausible responses to a cellular stress state. In this regard, Evans et al.^52^ observed increased PLA₂ activity in halibut (*Hippoglossus hippoglossus*) during embryonic development, accompanied by changes in phosphatidylcholine (PC) and phosphatidylethanolamine (PE) composition, culminating in reduced membrane fluidity.

On the other hand, the low-density (LD) group exhibited higher levels of LPC and LPE, lipids involved in signaling, repair, and membrane renewal processes^53^. The most affected LPC species included LPC 16:1, LPC 18:1, LPC 20:2, LPC 16:0, LPC 20:1, LPC 20:3, and LPC 18:2, all higher in LD, while LPC 14:0 was higher in HD. Among LPEs, LPE 18:1 and LPE 18:2 were also higher in LD. This profile may indicate a more dynamic metabolic state conducive to cellular plasticity, consistent with reduced cortisol release. Cortisol is a central modulator of lipid metabolism in fish, and its elevation under stress can promote lipid redistribution across energetic, structural, and signaling pathways^32^. Transcriptomic studies in *Micropterus salmoides* under high-density conditions demonstrate repression of lipid metabolism pathways mediated by PPARs and adipocytokines^54^, reinforcing the hypothesis of negative modulation of lipid remodeling under chronic stress. Additionally, observations in trout exposed to hypoxia and hyperoxia show that environmental stressors affect the expression of desaturases (FADS6D) and elongases, altering phospholipid saturation patterns^55^.

Altogether, the evidence suggests that stress resulting from high density may induce metabolic reprogramming directed toward membrane structural stability, with reduced phospholipid degradation and greater retention of specific PCs. While this pattern may help preserve cellular integrity, it may also limit membrane plasticity and signaling capacity. In contrast, low-density conditions appear to favor greater fluidity and remodeling capacity, indicating a more efficient metabolic balance. Although lipid oxidation was not directly assessed in this study, evidence in tilapia under high-density conditions indicates increased oxidative stress^9,56^, suggesting that the observed remodeling may also be related to antioxidant adjustments.

Finally, it is acknowledged that the present study had a relatively short duration (20 days) and evaluated only two stocking densities, which limits the extrapolation of the results and does not allow the determination of the optimal density range. Future studies should include long-term experiments covering the full growth cycle and multiple density gradients, as well as the integration of transcriptomic, metabolomic, and proteomic analyses to build a more comprehensive understanding of the metabolic networks involved in tilapia’s response to density-induced stress.

## Methods

The authors complied with the ARRIVE guidelines. This study was conducted in accordance with ethical principles for animal research and was approved by the Ethics Committee on the Use of Animals (CEUA) of the School of Veterinary Medicine and Animal Science, UNESP, Botucatu-SP campus (Protocol No. CEUA-0175/2018).

### Experimental design

The study was conducted at São Paulo State University – Júlio de Mesquita Filho (UNESP), School of Veterinary Medicine and Animal Science (FMVZ), Lageado Experimental Farm Campus, Botucatu, São Paulo, Brazil. Sixty-three sex-reversed male GIFT strain tilapia with an initial average weight of approximately 80 g were obtained from Trifish Aquaculture, Botucatu-SP. Fish were randomly assigned to six 0.42 m^3^ hapas (0.75 × 0.75 × 0.75 m) using a completely randomized design, ensuring homogeneous initial body weight across treatments. Three hapas were allocated for the high-density group (HD – 14 fish/hapa) and three for the low-density group (LD – 7 fish/hapa).

Fish underwent a seven-day acclimation period to the facilities and assigned densities prior to the trial commencement. During this period, fish were maintained under the same management as in the experimental phase, with continuous aeration, a natural photoperiod (12 h light:12 h dark), and daily monitoring of water quality parameters (temperature, pH, dissolved oxygen, and total ammonia), which remained within appropriate ranges for tilapia culture. During acclimation, fish were fed a commercial tilapia diet twice daily to apparent satiety.

### Diet and feeding

A pelleted diet containing 3000 kcal DE/kg and 29% digestible protein was formulated following the recommendation of the *“Brazilian Table for Nutrition of Tilapia”*^57^, which is specific for the growth of tilapia juveniles (Table 4). The fish were fed three times daily (9:00, 13:00, and 17:00) to apparent satiety.

**Table 4:** Chemical and nutritional composition of the diet.

Ingredients	Control (%)
Soybean meal-45	44.50
Soy protein concentrate	10.23
Poultry by-product meal	5.11
Corn, grain	33.80
Soybean oil	0.51
Wheat bran	2.05
DL-methionine	0.23
L-Threonine	0.37
L-Tryptophan	0.33
Dicalcium phosphate	2.06
BHT	0.02
Vitamin/mineral premix	0.61
Vit. C	0.09
NaCl	0.10
Total	100.00

Nutritional composition: dry matter (DM) 86.23%; gross energy (GE) 3,762 kcal/kg; crude protein (CP) 31.86%; digestible energy (DE) 3,044 kcal/kg; digestible protein (DP) 29.75%; crude fiber (CF) 3.31%; ether extract (EE) 3.03%; calcium (Ca) 0.81%; available phosphorus (*P* avail) 0.53%; and digestible energy to digestible protein ratio (DE/DP) 102.32. Average fatty acid profile of the diet (quadruplicate): C14:0 (0.09 ± 0.02), C16:0 (10.64 ± 1.08), C16:1n7 (0.09 ± 0.01), C17:0 (0.09 ± 0.00), C18:0 (2.76 ± 0.42), C18:1n9 (22.65 ± 2.97), C18:1n7 (0.09 ± 0.01), C18:2n6 (50.51 ± 6.27), C18:3n3 (7.29 ± 1.84), C20:1n11 (0.38 ± 0.02), C20:1n9 (0.30 ± 0.03), C20:5n3 EPA (0.08 ± 0.01), C22:1n11 (0.56 ± 0.07), C22:1n9 (0.22 ± 0.05), and C22:6n3 DHA (0.27 ± 0.03). *Source*: Author’s own data.

### Environmental parameters

The experiment was conducted with controlled air temperature. The hapas were installed in a 10,000-L tank with a constant water renewal rate of 5% per day and continuous aeration via porous stones connected to a central blower. Water temperature was maintained at approximately 27 °C through daily measurements at 17:00, with air temperature adjustments when necessary. To ensure water quality, samples from the recirculation system were collected every three days to determine pH, total ammonia, and dissolved oxygen levels, following established literature^58^. The photoperiod was maintained from 06:00 to 18:00, with lighting controlled by an automatic timer.

### Performance

Fish biometrics were performed at three different times: before the adaptation period, on the first day, and on the twentieth day of the experiment. Biometric values (initial body weight (IBW) and final body weight (FBW)) were used to calculate weight gain (WG) during the experimental period, obtained by subtracting the initial weight from the final weight. At the start of the experiment, six pre-tared containers, each containing 1000 g of feed (one container per hapa), were weighed. Apparent feed intake (AFI) was monitored daily per hapa by measuring the amount of feed consumed relative to the number of surviving fish. In case of mortality, the number of animals was updated for AFI calculation per hapa. At the end, the sum of daily intakes over the entire experimental period for each hapa resulted in three feed intake values per treatment.

Apparent feed conversion ratio (AFCR) was calculated by dividing the total apparent feed intake (AFI) by the average weight gain (WG) of the fish during the experimental period for each hapa, resulting in three AFCR values per treatment. Fillets were removed by a single trained fillet operator from both sides of the fish. Fillet yield (FY) was calculated by dividing the fillet weight by the FBW and multiplying by 100. After weighing, fillet samples were directed for further analyses.

### Fillet quality assessment

Fillet samples from 12 fish (six per treatment) were used for pH, color, proximate composition, texture, and fatty acid profile analyses. Fillet color was evaluated using a portable Chroma Meter CR400 colorimeter to measure L*, a*, and b* parameters according to Commission Internationale de l’Éclairage^59^, specifications on the right fillet portion after 24 h storage. Texture analysis was standardized on the right fillet side using a CT3-Brookfield texture analyzer (calibrated through pre-tests with experimental fillet samples). Deformation percentage was measured by compression on the cranial portion with a 71 g trigger load, 2 mm/s cylinder speed, and 20mm platform height. Proximate composition followed^60^, methods for moisture (39.1.02), protein (39.1.19), ether extract (39.1.05), and ash (39.1.09).

For fatty acid profiling, four right-side fillets per treatment were analyzed. Total lipids were extracted using cold technique^61^, followed by triacylglycerol transesterification^62^. Fatty acid quantification compared retention times against standard fatty acid methyl esters (Sigma Aldrich). Identification matched retention times with Sigma standards (USA), with peak areas determined using Clarity Lite v2.4.1.91 software. Quantification (mg g⁻^1^ total lipids) used methyl tricosanoate (Sigma) as internal standard.

### Lipidomic marker analysis

Frozen fillet samples were quickly crushed and homogenized to avoid thawing. They were then distributed into 2 mL microtubes (20 mg each) and subjected to extraction in different solvent systems according to the analyses performed: (A) 1 mL ethyl acetate solution^63^; (B) 1 mL chloroform/methanol 3:1 (v/v) solution^61^; and (C) methanol^64^. To maximize coverage of lipid classes with different polarities, these three extraction systems were selected, allowing a more comprehensive characterization of the tilapia lipidome. The microtubes were vortexed until complete homogenization and then sonicated in an ice bath for 30 min. Subsequently, the microtubes were centrifuged at 4,000 rpm for 5 min at 4 °C, and 250 μL of the supernatant were transferred to 1 mL glass vials for analysis.

For GC/MS analyses, the extracts obtained from solvent systems (A and B) were derivatized with BSTFA (N,O-Bis(trimethylsilyl)trifluoroacetamide) for 30 min at 70°C and injected (1.0 μL) into a 7990B gas chromatograph coupled to a 5977A mass spectrometer (Agilent Technologies), equipped with an HP-5 fused silica capillary column (5% diphenyl, 95% dimethylpolysiloxane). Helium was used as the carrier gas at a flow rate of 1 mL/min. Chromatographic separation used an injector temperature of 250 °C, transfer line temperature of 250 °C, and the ionization source operating in electron impact (EI) mode at 70 V. The mass spectrometer acquired data in full scan mode over a mass range of 35–300 m/z. The oven temperature program started at 40 °C for 2 min, followed by a 5 °C min⁻^1^ ramp to 150 °C, then 30 °C min⁻^1^ to 250 °C, held for 5 min.

Spectra elucidation was performed using the NIST/EPA/NIH Mass Spectral Library 2012 (included in the equipment software), linear temperature-programmed retention (LTPRI) determination, and other available literature libraries^65,66^. LTPRI determination involved a chromatographic run under the same parameters using an n-alkane standard mixture (C8–C20, Sigma-Aldrich).

Aliquots (1–5 μL) were injected into a hyphenated UHPLC system (Nexera X2, Shimadzu, Japan) equipped with an Acquity UPLC BEH C18 column (1.7 μm, 2.1 × 150 mm; Waters Technologies, USA) coupled to an Impact II mass spectrometer (Bruker Daltonics, Germany) with an electrospray ionization source operating in positive/negative modes and a Q-TOF analyzer. The UHPLC-MS/Q-TOF system was operated in full scan acquisition mode with data-dependent MS/MS for precursor ion and fragment identification. The high-resolution mass analyzer achieved a resolution greater than 40,000 FWHM at m/z 400, allowing precise molecular identification. The mass range analyzed was m/z 50–1200. The chromatographic gradient for the mobile phase consisted of a gradient mixture: solvent A (acetonitrile/water, 60:40, with 0.1% formic acid (v/v) or 10 mM ammonium acetate) and solvent B (isopropanol/acetonitrile, 90:10, with 0.1% formic acid (v/v) or 10 mM ammonium acetate). The chromatographic gradient was: 0 min, 15% B; 0–2 min, 30% B; 2–2.5 min, 48% B; 2.5–11 min, 82% B; 11–11.5 min, 99% B; 11.5–12 min, 99% B; 12–12.1 min, 15% B; 12.1–15 min, 15% B. The column temperature was maintained at 40 °C, and the flow rate was 0.2 mL min⁻^1^^64^.

### UHPLC-MS/MS data processing and compound annotation

MetaboScape v2023 (Bruker Daltonics, Bremen, Germany) was employed for processing *UHPLC-MS/MS raw data, including peak detection, chromatographic alignment, and compound identification *via* spectral library matching*. Annotation was performed using acceptance criteria of mass deviation (Δm/z) below 2 ppm and an mSigma value under 15, ensuring high accuracy in compound mass and isotopic pattern matching. MS/MS spectra were automatically matched against multiple spectral libraries, including Bruker HMDB 2.0, MassBank of North America (MoNA)^67^, LipidMaps^68^, LipidBlast^69^, MassBank Europe^70^, and METLIN^71^. All analyses were conducted in technical triplicates.

### Statistical analysis

The experimental design was completely randomized (CRD), with two treatments (high and low density) and three replicates (hapas) per treatment. The experimental unit varied according to the variable: for zootechnical parameters (AFI, AFCR), the experimental unit was the hapa (n = 3 per treatment), while for fillet quality and lipidomic analyses, the experimental unit was the individual (n = 4–6 per treatment, as detailed in the specific methods).

Statistical analyses were performed in R (version 4.3.1) using RStudio 2023.06.1 + 524. Data were tested for normality using the Shapiro–Wilk test. Comparisons between treatments were made using Student’s *t*-test. For univariate analyses of each lipid species, *p*-values were adjusted for multiple testing using the Benjamini–Hochberg method (FDR), applied within the significant biological classes.

For multivariate analyses, appropriate matrices were generated from the original abundance data. For univariate analyses, data were transformed using log(x + 1). For ordination and multivariate visualization (PCA), the Hellinger transformation was applied to relative abundances. The one-way PERMANOVA was performed using the adonis2 function from the vegan package on an appropriate dissimilarity matrix (Bray–Curtis). All tests were conducted at a significance level of α = 0.05.

## Conclusion

Stocking density markedly influences the productive performance, lipid metabolism, and fillet quality of juvenile Nile tilapia. Lower densities (LD) provide a less stressful environment, resulting in improved fillet yield and a more balanced lipidomic profile, characterized by enhanced phospholipid remodeling and higher concentrations of polyunsaturated fatty acids (PUFAs). However, these conclusions are based on a short-term trial with two density levels, which limits the assessment of chronic responses and the identification of an optimal density range. Future research should include long-term studies encompassing the entire production cycle and a broader range of stocking densities to determine threshold levels for welfare and performance. In addition, integrating physiological stress biomarkers (e.g., cortisol, oxidative stress indicators) with transcriptomic, proteomic, and metabolomic approaches would enable a deeper understanding of the molecular and metabolic mechanisms underlying density-induced stress. Such integrative analyses could provide valuable insights for developing management strategies that optimize fish welfare and productivity in intensive aquaculture systems.

## Data Availability

All data supporting this research are available for editors’ access at the following link: https://docs.google.com/spreadsheets/d/1Qzu0mRqhgouVN_hMRAB3ktbyH_-RDP8H/edit?usp=drive_link&ouid=111966773610880670816&rtpof=true&sd=true.
